# *Hepatozoon* spp. infection in wild canids in the eastern United States

**DOI:** 10.1186/s13071-023-05968-x

**Published:** 2023-10-19

**Authors:** Eliza Baker, Alex Jensen, Debra Miller, Kayla Buck Garrett, Christopher A. Cleveland, Justin Brown, Kyle Van Why, Richard Gerhold

**Affiliations:** 1 Department of Biomedical and Diagnostic Sciences, University of Tennessee College of Veterinary Medicine, Knoxville, TN USA; 2 Department of Forestry and Environmental Conservation, Clemson University Clemson, Clemson, SC USA; 3 Southeastern Cooperative Wildlife Disease Study, University of Georgia, Athens, GA USA; 4 Warnell School of Forestry and Natural Resources, University of Georgia, Athens, GA USA; 5 Department of Veterinary and Biomedical Sciences, College of Agricultural Sciences, The Pennsylvania State University, University Park, PA USA; 6 United States Department of Agriculture, Animal and Plant Health Inspection Service, Harrisburg, PA USA; 7 Center for Wildlife Health, University of Tennessee, Knoxville, TN USA; 8 One Health Initiative, University of Tennessee, Knoxville, TN USA

**Keywords:** American canine hepatozoonosis, Apicomplexan, *Hepatozoon americanum*, *Hepatozoon canis*, Parasitology

## Abstract

**Background:**

*Hepatozoon* spp. are apicomplexan parasites known to cause musculoskeletal disease in a variety of animals. Two species are known to infect wild and domestic canids in the US: *Hepatozoon canis* and *H. americanum.*

**Methods:**

In this study, blood, heart, and/or spleen samples were collected from 278 wild canids (180 coyotes, 93 red foxes, and 5 gray foxes) in the eastern US and tested via PCR for *Hepatozoon*. Histology slides of heart and skeletal muscle were assessed for *Hepatozoon* cysts and associated inflammation when fresh tissue was available (*n* = 96).

**Results:**

*Hepatozoon* spp. were found in 24.2% (59/278) of individuals, with *Hepatozoon canis* in 14.0% (34/278) and *H. americanum* in 10.7% (26/278). One coyote was positive for both *H. canis* and *H. americanum*. Foxes were more likely to be positive for *H. canis* than coyotes (23% and 7% respectively, *P* = 0.0008), while only coyotes were positive for *H. americanum.* Of the eight sampled states, *H. canis* was present in six (Louisiana, North Carolina, Pennsylvania, South Carolina, Tennessee, and Virginia) while *H. americanum* was found in two southern states (South Carolina and Louisiana). Infection status was positively correlated with myositis and myocarditis, and heart or muscle cysts were found in 83% (5/6) of *H. americanum*-positive coyotes.

**Conclusion:**

This survey showed a moderate prevalence of *H. canis* and *H. americanum* in states where the parasite was previously unrecorded including South Carolina and Pennsylvania.

**Graphical Abstract:**

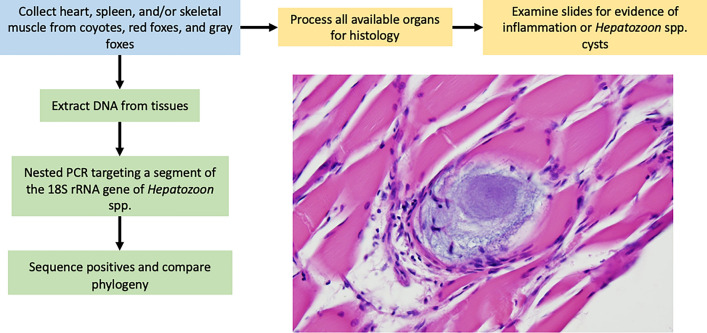

**Supplementary Information:**

The online version contains supplementary material available at 10.1186/s13071-023-05968-x.

## Background

*Hepatozoon* is a genus of apicomplexan parasites known to cause musculoskeletal disease in numerous terrestrial vertebrate species [1]. Hepatozoonosis is generally limited to the range of its tick definitive host, which varies by species and geographic area. Transmission occurs via ingestion of infected ticks or cystozoites in vertebrate hosts [2, 3]. In canids, there are two species are known to cause disease: *Hepatozoon canis*, the agent of Old World hepatozoonosis, and *H. americanum*, the agent of American canine hepatozoonosis (ACH) [2]. *Hepatozoon canis*, though common in South America, southern Europe, Asia, and Africa, was not documented in the US until 2008 [4]. Since then, it has been reported in seven southern states: Alabama, Georgia, Mississippi, Louisiana, Oklahoma, Virginia, and West Virginia [5, 6]. *Hepatozoon canis* typically causes mild disease in domestic dogs and may be discovered incidentally on blood smears or necropsy [2]. In patients with high levels of parasitemia, it can cause lethargy, fever, and anemia [2]. Little is known about transmission of *H. canis* in the US. The brown dog tick (*Rhipicephalus sanguineus)* is the accepted vector, though alternative methods of transmission may exist [6]. The Asian longhorned tick (*Haemaphysalis longicornis*), which has recently been introduced in the US, has been suggested as a possible competent vector for *H. canis* as well, and researchers have found oocysts in *H. longicornis* ticks pulled from dogs with hepatozoonosis [7, 8].

American canine hepatozoonosis is an emerging disease known to cause severe muscle pain, osteoproliferation, hyperesthesia, and death in domestic dogs [2, 9]. The parasite was first discovered in 1978 in coyotes (*Canis latrans)* from Texas [10]. ACH is restricted to the range of its vector, the Gulf Coast tick (*Ambylomma maculatum)*, which historically has been found in a 150-mile range around the Gulf of Mexico, ranging from southern Texas in the west to Virginia in the east [11]. However, in recent years, the tick has been documented as far north as Delaware and Illinois, and ACH has been documented as far from the Gulf as Vermont and California, though travel history for those cases in unknown [5, 12].

There are several suggested mechanisms contributing to the geographic expansion of the Gulf Coast tick and *H. americanum*, including the movement of vertebrate hosts and climate change. The transport of cattle for agriculture is responsible for the initial expansion of *A. maculatum* to the central US [13]. Migratory birds, which are known to host larval *A. maculatum* ticks, may also play a significant role in its spread [14]. In addition, climate modeling predicts that the tick’s range will continue to expand northward as the climate warms, with suitable habitats predicted to extend as far north as Maine [15].

The role of wild canids in the epidemiology of ACH is poorly understood. Investigations into wildlife reservoirs have found a high prevalence in coyotes in the southern US. For example, a 2013 study in Oklahoma and Texas showed a prevalence of 79.5% (35/44) when examining histology, whole blood PCR, and muscle PCR of the 18S ribosomal RNA gene [16]. Genetic diversity was high, with 19 distinct sequences and up to seven different haplotypes infecting an individual coyote. Only one *H. canis* infection was found, and the remaining genotypes were either *H. americanum* or an intermediate species between the two.

Coyotes appear more resistant to clinical disease than domestic dogs, though experimental infections have shown they can develop osteoproliferative lesions similar to dogs [17]. Investigations into wild canids in areas outside of the south-central US are limited, and coyotes’ role in the life cycle is unclear. Some researchers believe that coyotes act as important wildlife reservoirs for the disease, while others believe both wild and domestic canids are accidental hosts, and there is an unknown intermediate host that serves as the primary reservoir [2]. Regardless, further research is necessary to understand the spread and potential impact of this disease on both domestic dogs and wild canids in the US. We conducted a prevalence survey of wild canids throughout the eastern US, a region that has not been assessed for this pathogen on a wide scale since 2008 [5]. We hypothesized that *H. americanum* would be prevalent in southern states and absent in the north and that *H. canis* would be found rarely in both foxes and coyotes. In addition, we hypothesized that infected canids would have higher rates of myositis and myocarditis compared to negative canids.

## Methods

We opportunistically collected a total of 278 coyote, red fox (*Vulpes vulpes*), and gray fox (*Urocyon cinereoargenteus)* tissue samples from a variety of sources and collaborators including rabies-testing facilities, road-killed animals, and wildlife resources agencies (Table 1). Whole blood and/or heart tissue was collected between 2019 and 2023 from wild canids in South Carolina (*n* = 59), Tennessee (*n* = 73), and Virginia (*n* = 15), and splenic samples were collected from wild canids between 2021 and 2022 from Louisiana (*n* = 27), Pennsylvania (*n* = 92), Georgia (*n* = 3), North Carolina (*n* = 7), and Maryland (*n* = 2). We obtained 94 whole carcasses in Tennessee (*n* = 71), South Carolina (*n* = 15), and Virginia (*n* = 8). Heart tissue only was available from two Tennessee coyotes.

**Table 1:** Prevalence of *Hepatozoon canis* and *H. americanum (H. am)* in foxes and coyotes from the eastern US based on PCR of heart, blood, and/or spleen

State	Fox			Coyote		
	Samples	*H. canis (%)*	*H. am (%)*	Samples	*H. canis (%)*	*H. am (%)*
GA	Spleen (*n* = 3)	0	0	NA	–	–
LA	Spleen (*n* = 2)	1 (50)	0	Spleen (*n* = 25)	2 (8)	3 (12)
MD	Spleen (*n* = 1)	0	0	Spleen (*n* = 1)	0	0
NC	Spleen (*n* = 3)	0	0	Spleen (*n* = 4)	1 (25)	0
PA	Spleen (*n* = 82)	17 (21)	0	Spleen (*n* = 0)	7 (70)	0
SC	NA	–	–	Heart (*n* = 9)	0	2 (22)
				Blood (*n* = 59)	2 (3)	23 (39)
				Histology (*n* = 15)	0	5 (33)
				Total SC coyote (*n* = 59)	2 (3)	23 (39)
TN	Heart (*n* = 5)	1 (20)	0	Heart (*n* = 61)	0	0
	Blood (*n* = 1)	1 (100)	0	Blood (*n* = 68)	0	0
	Histology (*n* = 5)	0	0	Histology (*n* = 66)	0	0
	Total TN fox (*n* = 5)	1 (20)	0	Total TN coyote (*n* = 69)	0	0
VA	Heart (*n* = 2)	2 (100)	0	Heart (*n* = 13)	1 (8)	0
	Blood (*n* = 2)	1 (50)	0	Blood (*n* = 13)	1 (8)	0
	Total VA fox (*n* = 2)	2 (100)	0	Histology (*n* = 8)	0	0
				Total VA coyote (*n* = 13)	1 (8)	0
Total	Total fox (*n* = 98)	21 (21)	0	Total coyote (*n* = 180)	13 (7)	26 (14)

The presence of cysts on histology, when available, is also shown. Total number positive is listed with percent positive in parentheses

DNA was extracted from 100 µl of whole blood (*n* = 141), 10 mg of spleen (*n* = 131), and/or 25 mg of heart (*n* = 90) using DNeasy Blood and Tissue extraction kits following manufacturer’s instructions (Qiagen Inc, Germantown, MD, USA). A negative water control was used during each extraction. PCR was performed using nested PCR primers targeting the 18S rRNA gene of all *Hepatozoon* spp. and other closely related apicomplexans, using both negative extraction and negative PCR controls [4, 18]. Primers 5.1 (CCTGGTTGATCCTGCCAGTAGT) and 3.1 (CTCCTTCCTTTAAGTGATAAG) were used for the external reaction. Primary cycling conditions were as follows: initial denaturation for 5 min at 95 °C followed by 35 cycles of 94 °C for 1 min, 56 °C for 1 min, and 72 °C for 1.5 min, and a final annealing step of 72 °C for 7 min. The internal reaction used primers RLB-F (GAGGTAGTGACAAGAAATAACAATA) and RLB-R (TCTTCGATCCCCTAACTTTC) and 1 µL of the primary product. The secondary reaction cycling conditions were the same as the primary except the annealing temperature was lowered to 52 °C. Extracted DNA from an *H. americanum*-positive dog was used as the positive control. PCR products were visualized on 1.5% agarose gel, and amplicon bands between 550 and 570 bp were purified with ExoSAP-IT PCR Product Cleanup Reagent (Thermo Fisher Scientific, Waltham, MA) and sequenced with Sanger sequencing at the University of Tennessee, Knoxville’s Division of Biological Sequencing. Amplicon sequences were analyzed in Sequencher v. 5.4.6 (Gene Codes Corp., Ann Arbor, MI), and those with multiple sequences were cloned using the pGEM-T Easy Vector System (Promega Corporation, Madison, WI) following manufacturer’s instructions. Plasmids were purified using MiniPrep Plasmid Purification kits (Thermo Fisher Scientific, Waltham, MA) following manufacturer’s instructions. Sequences were deposited in GenBank under accession numbers OQ592065–OQ592143. Sequences were aligned in BioEdit, and phylogenetic trees were made using the neighbor-joining algorithm with the Kimura 2-parameter model with 500 bootstrap replicates in MegaX (v10.1.7) [19]. We removed identical sequences to improve the readability of the tree.

We examined histopathology slides from tongue, heart, and gracilis muscle when tissue was available (*n* = 96), with assistance from a veterinary pathologist (author DM). We classified the inflammation as mild if it was found rarely throughout the slide and did not disrupt surrounding tissues and moderate if it disrupted the surrounding tissues but was present only focally or multifocally. We considered the inflammation severe if it was found throughout the slide and disrupted the surrounding tissue. Statistical analysis was performed using SAS v9.4 (SAS Institute, Cary, NC, USA). Chi-squared tests were used to assess statistical differences among species, infection status, and histopathological inflammation.

## Results

Sixty-five percent of samples were from coyotes (*n* = 180) and 35% from foxes (93 red foxes and 5 gray foxes). *Hepatozoon* spp. were detected via PCR in 21% (59/278) of the individuals tested; 9% (26/278) were positive for *H. americanum* and 12% (34/278) for *H. canis* (Table 1). One coyote from South Carolina was co-infected with both species. All canids with cysts present on histology were also positive on PCR of blood or tissue. *Hepatozoon americanum* was exclusively found in coyotes, where it was present in 14% (26/180) of canids. *Hepatozoon canis* was found in both coyotes (7%, 13/180) and red foxes (22.5%, 21/93). All gray foxes were negative for *Hepatozoon* spp. (*n* = 5). The likelihood of *H. canis* infection in red foxes was significantly higher than in coyotes (*X*^2^ = 14.2, df = 1, *p* = 0.0008).

**Fig. 1:**
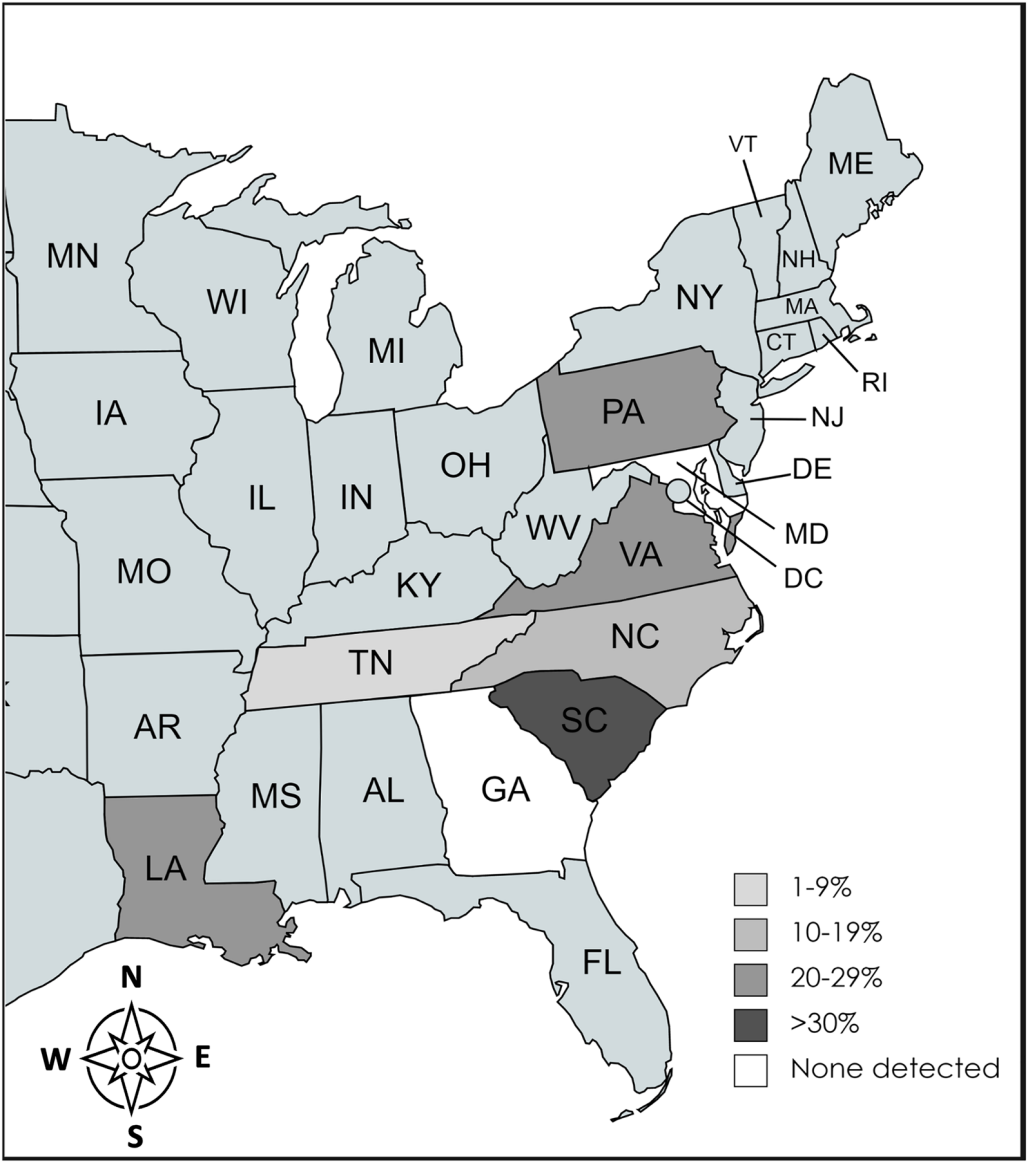
Prevalence of *Hepatozoon* spp. detected via PCR of heart, blood, and/or spleen in wild canids in the eastern USA Created with MapChart

Infections with any species of *Hepatozoon* were identified in six of the eight tested states (Fig. 1). *Hepatozoon americanum* was only found in two of the tested states: South Carolina (39% [23/59] of coyotes) and Louisiana, (12% [3/25] of coyotes). *Hepatozoon canis* was found in all tested states except for Georgia and Maryland (Table 1). However, these two states had fewer than four individuals each, making assessment of prevalence impossible. Paired blood and tissue samples were available for 86 of the canids. Blood and tissue PCR agreed 99% of the time, with only one canid from Virginia positive for *H. canis* on heart but negative on blood.

Phylogenetic alignment of the 18S rRNA gene performed using one sequence for each unique sequence group, along with related organisms and *Adelina bambarooniae* (AF494058) as the outgroup, resulted in a 488-bp alignment of which 390 were invariant and 32 of the 98 variable characters were parsimony informative. The top match in GenBank for each unique sequence was included in the tree for comparison. Sequence analysis of the *Hepatozoon* sequences revealed 34 unique sequences. Cloning was performed on 11 PCR products, with up to four unique sequences present within an individual sample. One coyote was co-infected with both *H. americanum* and *H. canis.*

*Hepatozoon americanum* sequences were between 94.2 and 100% similar to each other, with 46% (21/45) of *H. americanum* sequences > 99.5% similar to several sequences from dogs (e.g. EU146062 and AY864676) and coyotes (e.g. JX415170-JX415174) in the southern US. We found a second group of sequences (*n* = 11) that aligned more closely with *H. americanum* than *H. canis* but did not fully resolve into the *H. americanum* clade. These sequences most closely aligned with several sequences in GenBank including a South American gray fox (*Lycalopex griseus*) from Argentina (MK049949) and a Pampas fox (*Lycalopex gymnocercus*) from Uruguay (MZ230033). We found minimal genetic diversity in the *H. canis* sequences, with sequences 98.2–100% similar to each other and 79% (27/34) of sequences aligning > 99.5% with several sequences from around the world including foxes from France (MK673844-MK673850), gray wolves from Germany (MN791089), and a dog from Cuba (MN393911)*.* We found another distinct genotype of *H. canis* in five canids from Virginia, Tennessee, and North Carolina (OQ592106-OQ592110), which aligned 99.78% with several sequences from around the world including dogs from Thailand (MK830996), Algeria (MK645969), Nigeria (OP837324), and India (JN584477). Two Louisiana canids (OQ592117 and OQ592118) aligned 100% with a coyote from Oklahoma (JX415165). Unlike *H. americanum,* we did not find any evidence of paralogs or coinfection with multiple genotypes of *H. canis.*

**Fig. 2:**
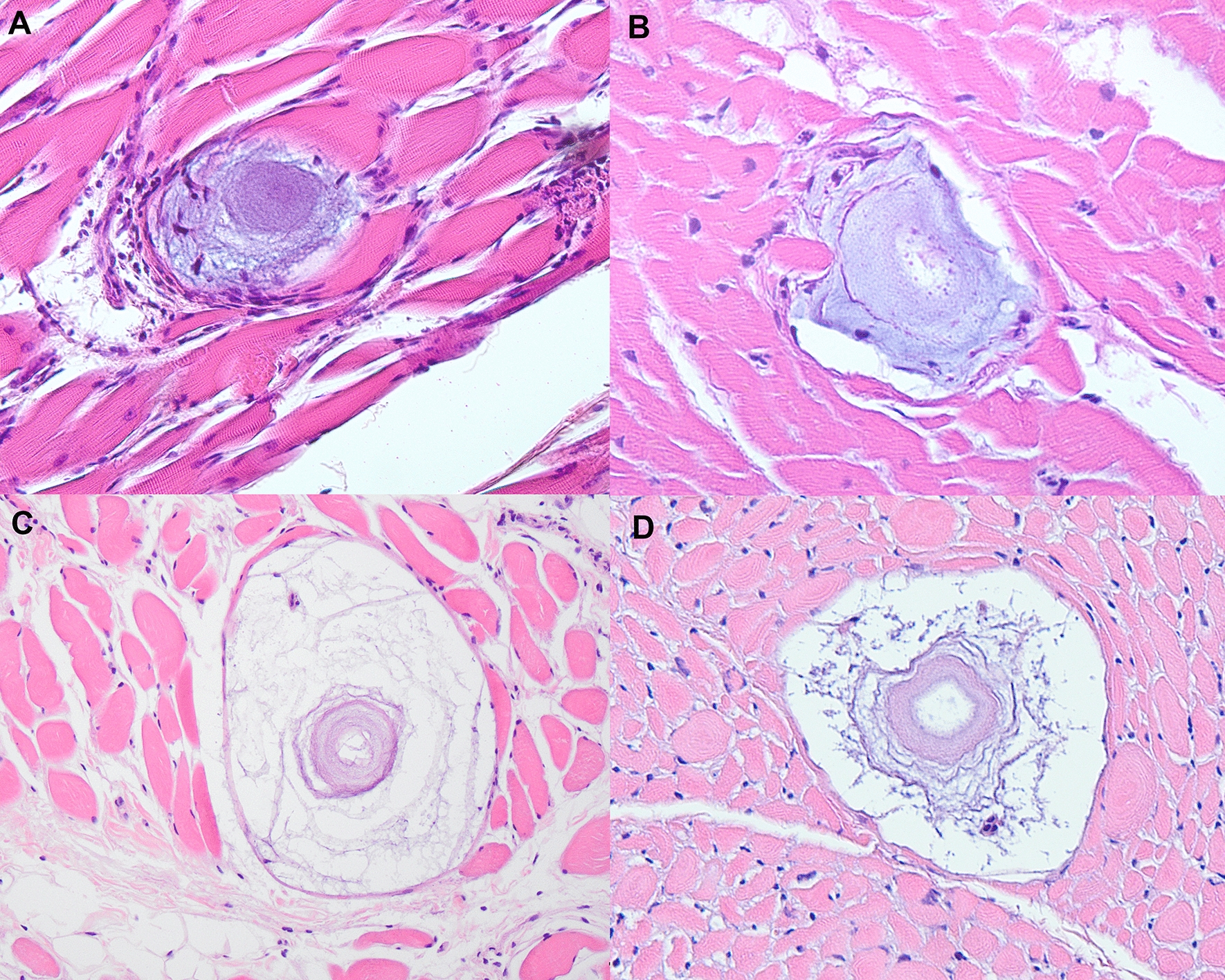
Histology images of cysts in various stages of development from coyotes positive for *Hepatozoon* spp. collected in the eastern US. *H. americanum* cysts were present in the gracilis muscle (**A**, **D**), heart (**B**), and tongue (**C**)

Histopathology was processed from 96 of the 248 total samples. Heart tissue was processed for 91 coyotes and 5 foxes; gracilis muscle was processed for 89 coyotes and 4 foxes; tongue was processed for 37 coyotes and 4 foxes; spleen was processed for 89 coyotes and 4 foxes. Cysts were found in the heart or skeletal muscle of five coyotes, all of which were positive for *H. americanum* on PCR (Fig. 2). Eighty-three percent (5/6) of the *H. americanum*-positive specimens with histopathology had at least one cyst present in heart or skeletal muscle. Cysts were present by histology in both heart and skeletal muscle in two coyotes, in skeletal muscle alone in two coyotes, and in heart alone in one coyote. The *H. canis*-positive fox did not have cysts present on histology.

**Fig. 3:**
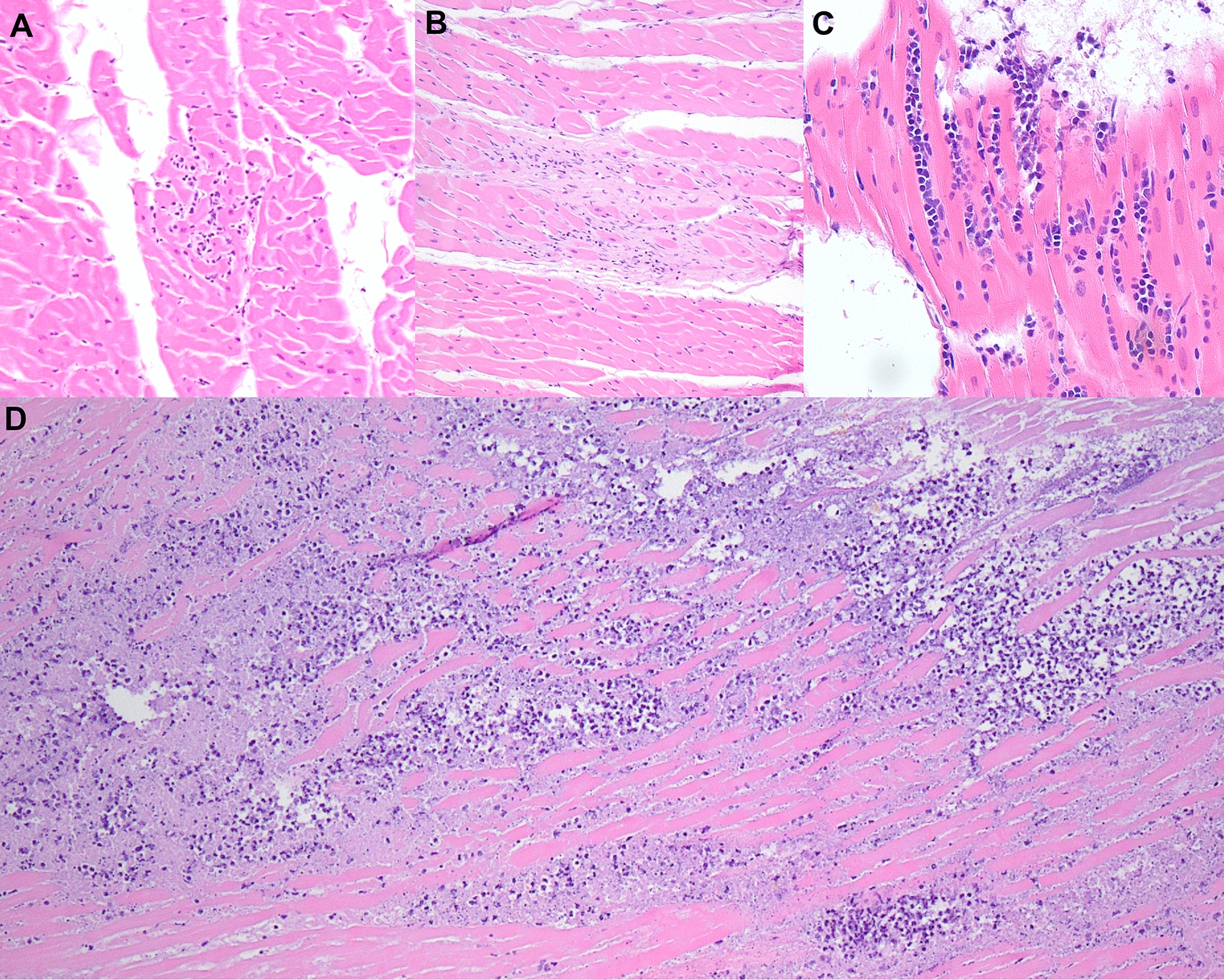
Histology images from *Hepatozoon*-positive coyotes showing examples of **A** mild, **B** moderate, and **C** severe myocarditis. The overwhelming suppurative myocarditis found in the red fox infected with both *Hepatozoon canis* and sarcoptic mange is shown in **D**

Two of the *Hepatozoon*-positive individuals had severe pathology. The *H. canis*-positive red fox also had sarcoptic mange that resulted in sepsis and severe suppurative myocarditis (Fig. 3). One *H. americanum*-positive coyote had a regionally extensive abscess beneath its esophagus extending into the cervical bone and causing osteomyelitis. Evidence of sepsis and bacterial embolisms were present throughout most major organs. This coyote had the largest number of *Hepatozoon* cysts, with ten cysts in various stages of development found throughout the tongue and occasional cysts found in the gracilis muscle and heart. In addition, two of the positive coyotes had limb abnormalities. One coyote was missing its back left leg entirely after the mid-diaphysis of the femur, while the other had a shrunken back left leg about half the size of the right hind leg with the diaphysis of the femur replaced by dense connective tissue. The femur measured 7 cm.

Myocarditis was present in 8.3% (8/96) of samples, and myositis was present in 10.8% (10/93) (Fig. 3). The prevalence of myocarditis was statistically higher in *Hepatozoon* spp.-positive canids than negative individuals (*X*^2^ = 23.5, df = 1, *p* < 0.001), with myocarditis present in 57% (4/7) of positive cases compared to 4.5% (4/89) of negative cases. However, two myocarditis cases occurred in animals suffering from sepsis (the fox with sarcoptic mange and the coyote with osteomyelitis). Therefore, we re-assessed the statistical correlation between myocarditis and *Hepatozoon* status with those two cases removed, and the correlation remained (*X*^2^ = 21.2, df = 1, *p* = 0.002). Likewise, myositis was found in 50% (3/6) of positive cases and only 8% (7/87) of negative cases (*X*^2^ = 10.3, df = 1, *p* = 0.001). Skeletal muscle was not available from the sarcoptic fox or from two of the Tennessee coyotes. Both myositis and myocarditis were typically mild to moderate apart from the two septic animals (Fig. 3).

## Discussion

*Hepatozoon* spp. infections are widespread in wild canids throughout the eastern US. *Hepatozoon canis* was detected in six states in our study, while *H. americanum* was restricted to only two states in the south despite evidence that *A. maculatum* has spread as far north as Delaware [12]. Both foxes and coyotes were infected with *H. canis*, but *H. americanum* was detected exclusively in coyotes, potentially because of the low number of foxes sampled in the southern states. To the authors’ knowledge, this is the first report of *H. canis* or *H. americanum* in South Carolina and Pennsylvania. Though evidence of infection was not found in two of the eight states (GA and MD), only three samples were collected from Georgia and only two from Maryland, making critical interpretation of prevalence in those states impossible.

There are limited studies on *Hepatozoon* spp. prevalence in the US with which to compare our findings. A 2011 study published *Hepatozoon* spp. sequences from a variety of mammals in the southern US, including five coyotes with *H. americanum* from Oklahoma and Texas and one gray fox with *H. canis* from Georgia [20]. Like our study, individual coyotes often had multiple different sequences of *H. americanum*. There are only two other surveys assessing prevalence in coyotes, both of which focused on the south-central US. One survey reported *Hepatozoon* spp. prevalence of 40% in 20 coyotes in Oklahoma via histology, and a second study reported a prevalence of 80% in 44 coyotes in Oklahoma and Texas using a combination of histology and PCR [16, 17]. Prevalence in the eastern US was substantially lower. The cause for this lower prevalence is not fully clear. Coyotes only recently migrated across the eastern US, beginning in the mid-1900s and not reaching the east coast until the late 1900s [21]. Although it is possible that *H. americanum* emerged in the east alongside this migration, further research is necessary to determine if the eastern migration of coyotes has impacted *H. americanum* prevalence.

A 2008 survey of *Hepatozoon* in the US included samples from 614 domestic dogs with suspected hepatozoonosis, 455 of which were from the southeast [5]. It found *H. americanum* in 13 of 28 states, and 5 states had both *H. americanum* and *H. canis*. Unlike in our study, *Hepatozoon* was not found in Tennessee or Pennsylvania, and samples from South Carolina were not obtained. Positive cases were primarily found in the southeastern US, though outliers with unknown travel history in Vermont, Washington, and California were detected [5].

Our study found a higher rate of *H. canis* than the domestic dog survey [5]. Only 4.6% (28/614) of domestic dogs were positive for *H. canis* compared to the 12.4% (34/278) prevalence we found in wild canids. The relative percentages of each *Hepatozoon* species were also markedly different between domestic dogs and our study. Only 14.4% of *Hepatozoon* cases in domestic dogs were *H. canis* or mixed infections, while 57% of cases in wild canids from our study were *H. canis* or mixed infections*. H. canis* infections were rare in the Texas and Oklahoma coyote study as well, with only 2% (1/44) of coyotes positive [16]. The high prevalence of *Hepatozoon canis* in our study is likely explained by our inclusion of foxes, which had a significantly higher prevalence than dogs or coyotes. Whether *H. canis* is more common in the east than the central US remains to be seen, and future work assessing foxes in that area may clarify the question. Though the domestic dog study found a higher prevalence of *H. americanum* than we did (29.4% vs. 10.4%), a direct comparison cannot be made since the dog survey targeted suspected positive dogs, while ours sampled all individuals [5].

Despite the severe clinical signs of ACH in domestic dogs, infection did not appear to cause significant lesions in coyotes. However, infection status was positively correlated with risk of myocarditis and myositis. Myositis was six times more likely in positive individuals, while myocarditis was over ten times more likely. However, with only seven positive cases processed for histopathology, two of which had confounding factors, further research is necessary to determine the strength of this correlation. Even if the correlation persists in larger surveys, a causal connection could not be established from surveys alone given that *Hepatozoon* spp. infection is commonly associated with co-infections that may be the true cause of inflammation [22–25].

Though two of the six *H. americanum*-positive coyotes had limb abnormalities, this is not likely a result of ACH. ACH is known to cause osteoproliferation and musculoskeletal pain, but loss of limbs or severe limb deformities are not known symptoms. In addition, the coyote with the missing hind limb survived at least 1.5 years after initial sampling and was in good body condition at the time of necropsy. It is possible that *Hepatozoon* infection can be exacerbated by immunosuppression. Domestic dogs typically only develop clinical signs from *H. canis* if they are immunosuppressed or co-infected with other diseases like *Babesia, Leishmania*, or *Toxoplasma gondii* [2]. The coyote with sepsis had notably increased numbers of *Hepatozoon* cysts compared to all other positives, suggesting that replication may increase during times of stress or illness. The *H. canis*-positive red fox with sarcoptic mange unfortunately only had heart, skin, and kidney processed for histopathology, none of which are the typical locations to find meronts. Therefore, though we did not find meronts, we cannot assess the extent of its *H. canis* infection.

**Fig. 4:**
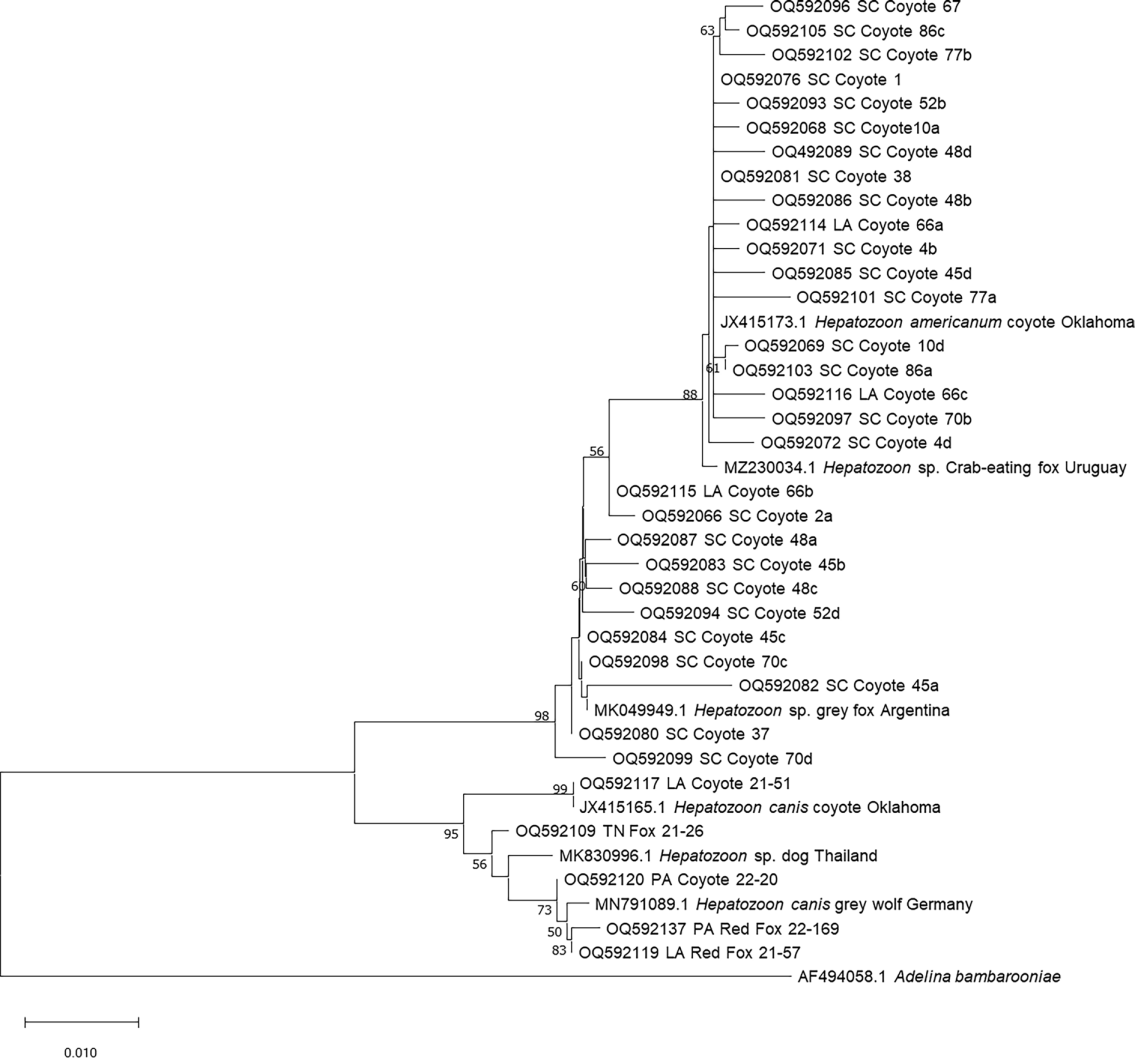
Phylogeny of the partial 18S  rRNA gene of *Hepatozoon* spp. found in wild canids in the eastern US. *Adelina* was chosen as the outgroup. All unique sequences are shown. The phylogenetic tree was generated using the neighbor-joining algorithm and a Kimura 2-parameter model in MegaX with (v10.1.7). The percentage of replicate trees in which the associated taxa clustered together in the bootstrap test (500 replicates) are shown next to the branches

We found variable levels of genetic diversity (94.2%–100% similar) in the *H. americanum*-positive coyotes. Forty-two percent of the positive coyotes (11/26) had evidence of multiple different sequences of *H. americanum* present. Previous work on *Hepatozoon* in coyotes suggested these different sequences were evidence of coinfection with multiple related *Hepatozoon* species, but intraspecific variation of the 18S gene (paralogs) have been documented in *H. canis* and numerous other apicomplexan species [16, 20, 26]. Therefore, we cannot definitively say which is the case in these samples. Though most sequences fully resolved into the *H. americanum* clade, a subset fell in between *H. canis* and *H. americanum* (Fig. 4). Similar sequences were found previously in coyotes from Texas and Oklahoma [16].

We found lower genetic diversity (98.2–100%) in our *H. canis* sequences compared to *H. americanum*, with most sequences differing by fewer than three base pairs. This limited diversity has been documented in previous work, which classified *H. canis* 18S rRNA sequences into five genotypes, with each genotype differing by five base pairs or fewer [27]. A larger survey assessing various haplotypes of the 18S gene of *H. canis* worldwide found 76 total haplotypes [28]. However, of the 61 sequences they assessed from the Americas, they found > 50% of them differed by fewer than two base pairs, matching the results from our study. It is interesting that three different *H. canis* genotypes were documented in southern states while only one genotype was documented in the north. This suggests diversity may be higher in the south, although further research is necessary to verify this assessment.

This study provided a clearer understanding of the geographic distribution of *Hepatozoon* spp. in the eastern US. Wild canids appear to play a significant role in maintaining the sylvatic life cycle of *Hepatozoon*. A high prevalence in wildlife implies a high percentage of infected ticks in the area and can be used as a proxy for domestic dog risk. In addition, *Hepatozoon* prevalence can provide a better understanding of its vectors’ prevalence in the area. The tick vectors of *Hepatozoon* may carry other pathogens including *Ehrlichia canis (R. sanguineus), Babesia canis vogeli (R. sanguineus), Francisella tularensis (A. maculatum), Rickettsia parkeri* (*A. maculatum*), and *Rickettsia rickettsii (R. sanguineus)* [15, 29–32]*.* These pathogens may lead to severe disease in dogs on their own or contribute to the severity of coinfections, and both *Rickettsia* spp. pathogens can cause life-threatening disease in people [29, 32]. Though hepatozoonosis is still considered rare in domestic dogs, the high prevalence in wild canids suggests a high potential rate of exposure, and the disease should be among the differentials for dogs presenting with muscle pain, fever, and neutrophilia.

Limitations of this study include the opportunistic nature of collection, the limited sample size in certain states, and the variability of available tissue samples from individual canids. Future work can help fill in the geographic gaps of this survey to better understand the distribution and impact of this pathogen. In addition, future studies should strive to use whole carcasses for analysis whenever possible to give a clearer picture of tissue tropisms and tissue responses. Though our study found a high agreement between histology and PCR results, a previous study showed disagreement between the two methods, and the gold standard for diagnosis is still considered muscle biopsy [2, 16]. Therefore, assessment of *Hepatozoon* spp. prevalence ideally would always include both histology and PCR. Further research is necessary to assess this disparity, though PCR of whole blood may be a reasonable, less invasive alternative diagnostic tool in cases where obtaining a muscle biopsy is not practical. Finally, phylogenetic analysis using only a small section of the 18S rRNA gene may miss significant variation found elsewhere in the gene. Future studies can improve our understanding of canid *Hepatozoon* genotypes by using longer sequences or full genome sequencing.

## Conclusion

*Hepatozoon* is a widespread parasite infecting wild canids throughout the eastern US. *Hepatozoon canis* is far more common, particularly in foxes, than previously realized. Mild inflammation in both the skeletal muscle and the heart was more likely in infected individuals (Additional file 1: Table S1).

### Supplementary Information

**Additional file 1: Table S1.** List of species, capture locality and PCR results. Number of cysts listed for positive cases with available histopathology.

## Data Availability

All necessary data is provided within the text or tables of this manuscript.

## Abbreviation

ACH: American canine hepatozoonosis
